# Intercropped Silviculture Systems, a Key to Achieving Soil Fungal Community Management in Eucalyptus Plantations

**DOI:** 10.1371/journal.pone.0118515

**Published:** 2015-02-23

**Authors:** Caio T. C. C. Rachid, Fabiano C. Balieiro, Eduardo S. Fonseca, Raquel Silva Peixoto, Guilherme M. Chaer, James M. Tiedje, Alexandre S. Rosado

**Affiliations:** 1 Institute of Microbiology Paulo de Góes, Federal University of Rio de Janeiro, Rio de Janeiro, Rio de Janeiro, Brazil; 2 Embrapa Solos, Embrapa, Rio de Janeiro, Rio de Janeiro, Brazil; 3 Embrapa Agrobiologia, Embrapa, Seropédica, Rio de Janeiro, Brazil; 4 Center for Microbial Ecology, Michigan State University, East Lansing, Michigan, United States of America; Leibniz Institute DSMZ-German Collection of Microorganisms and Cell Cultures, GERMANY

## Abstract

Fungi are ubiquitous and important contributors to soil nutrient cycling, playing a vital role in C, N and P turnover, with many fungi having direct beneficial relationships with plants. However, the factors that modulate the soil fungal community are poorly understood. We studied the degree to which the composition of tree species affected the soil fungal community structure and diversity by pyrosequencing the 28S rRNA gene in soil DNA. We were also interested in whether intercropping (mixed plantation of two plant species) could be used to select fungal species. More than 50,000 high quality sequences were analyzed from three treatments: monoculture of *Eucalyptus*; monoculture of *Acacia mangium*; and a mixed plantation with both species sampled 2 and 3 years after planting. We found that the plant type had a major effect on the soil fungal community structure, with 75% of the sequences from the Eucalyptus soil belonging to Basidiomycota and 19% to Ascomycota, and the Acacia soil having a sequence distribution of 28% and 62%, respectively. The intercropping of *Acacia mangium* in a *Eucalyptus* plantation significantly increased the number of fungal genera and the diversity indices and introduced or increased the frequency of several genera that were not found in the monoculture cultivation samples. Our results suggest that management of soil fungi is possible by manipulating the composition of the plant community, and intercropped systems can be a means to achieve that.

## Introduction

Planted forests represent a cheap and renewable source of raw material for industry and reduce the pressure on native vegetation [1]. These forests are an important component of the economy in many countries. In Brazil, 6.5 million hectares of forest is cultivated, playing an important economic role and a source of employment for millions of citizens. *Eucalyptus* and *Pinus* are the genera most commonly used for silviculture in Brazil and are the most important sources of wood, cellulose and biochar (biofuel) for industry. *Acacia mangium* and *A*. *mearnsii* were more recently introduced to the same uses, in addition to tannin extraction and rehabilitation of degraded areas [2–4].

However, most tree plantations are monocultures; i.e., in Brazil, the planted forests are mainly composed of monocultures of *Eucalyptus sp*. [2], which can result in a lower ecosystem stability, with higher occurrence of pathogens and pests [5,6] and nutrient disorder/imbalance [6,7].

Alternative management methods are being developed with the utilization of legume trees in association with *Eucalyptus* in mixed stands, with the aim of improving the system biodiversity, soil quality and wood productivity [8–19]. Studies have revealed that intercropped plantations (compared with monoculture) have the potential to increase biomass productivity, efficiency of light use, soil nitrate content, soil C and N stocks, P release and deposition, and the quality and decomposition of the litter.

Fungi are ubiquitous organisms of soil and important components in silviculture. They are important for soil nutrient cycling, with a vital role in C, N and P turnover, and their relation with plants can vary from pathogen to mutualist. Through mycorrhizal associations, they can promote plant development, improving nutrient uptake and confer stress resistance and protection against pathogens. Mycorrhizal associations are found in more than 80% of plants, and most of them are dependent on these associations for efficient nutrient uptake [20]. Studies have demonstrated an increase in both *Eucalyptus* and *Acacia* development when associated with mycorrhizal fungi [21,22]. These trees can establish associations with both ectomycorrhizae and arbuscular mycorrhizae [21,23,24].

In a previous study [25], our group investigated the changes in soil chemistry and their relation to the microbial community in mixed and pure plantations of *Acacia mangium* and *Eucalyptus urograndis* in a field experiment. Using DGGE and real-time PCR, the structure and the abundance of different microbial groups and nitrogen cycling genes were screened. The results revealed a snapshot of the communities, and a clear effect of the treatments on the microbial community was detected, highlighting the need to better understand the impact of soil chemistry and plant species.

To address this question, our goal was to evaluate the soil fungal community using pyrosequencing over two consecutive years. We were especially interested in describing the fungal diversity in the forestry system and the influence of tree composition on the community. Our hypotheses were: i. the soil fungal community is influenced by plant species; ii. the soil fungal community changes with time; and iii. the mixture of tree species promotes the mixing of the soil fungal community. Our data revealed a strong influence of the plant species on the soil fungal community and showed that the management of soil fungi is possible by manipulating the plant community.

## Materials and Methods

### Ethics statement

The samples were collected in a non-protected area and did not involve endangered or protected species according to Brazilian laws. The experiment was conducted in an experimental field of the research institution Embrapa Agrobiologia, in Seropédica, Rio de Janeiro State, Brazil (22^o^ 46^’^ S; 43^o^ 41^’^ W; 33 m altitude).

### Site description

A complete site description can be found in Rachid el al. 2013 [25]. Briefly, the experiment was established in an area that was left fallow for more than 15 years and was covered by grasses of natural occurrence. The soil is classified as Haplic Solonetz according to the FAO/UNESCO system of soil classification (Planosolo háplico, according to the Brazilian Soil Taxonomy), characterized by sandy topsoil (~90% sand), low cation exchange capacity (CEC), and low organic matter and nutrient contents. The climate of the study area is classified as Aw (tropical with a dry winter). The average annual precipitation is 1,250 mm, the mean daily air temperature ranges from 16°C (June and July) to 32°C (January to March), and the mean relative air humidity is 73%.

### Experimental design

The experimental design was previously described in Rachid el al. 2013 [25]. A randomized block design was used, with three treatments and four replicates. The treatments included monospecific stands of *Eucalyptus urograndis*, hereafter called Eucalyptus; monospecific stands of *Acacia mangium*, hereafter called Acacia; and an intercropped plantation of these two species, hereafter called Mix. The tree seedlings were planted in 3 m × 3 m spacing in 18 m × 21 m (378 m^2^) plots in January 2009.

To avoid a ‘border effect’, a useful sampling area was established within each plot by excluding two rows of trees on all four sides of each plot. This resulted in a sampling area comprising 12 central trees.

Before planting operations, the vegetation in the entire experimental area was mowed a week after glyphosate application. Soil correction was made with the application of two Mg ha^-1^ of lime. Fertilization was performed during the planting operations by adding 100 g of P_2_O_5_ (super triple phosphate), 40 g of K_2_O (KCl) and 25 g of fritted trace elements (FTE) to the planting hole of each seedling. The pure Eucalyptus stands also received 20 g of N (ammonium sulfate) at planting and additional doses of 20 g at 60, 160, and 300 days after planting. Nitrogen fertilization in the Eucalyptus plantation followed what is normally found in commercial plantations. Nitrogen fertilization was not performed in the Mix or Acacia treatments, in order to test if the legume tree was able to supply nitrogen to the system [25].

### Sampling

Each treatment was represented by four composite samples per year (eight replicates in total). The soil was sampled in December 2010 and December 2011 (summer) when the stands were 2 and 3 years old. Five single samples (0–10 cm, collected halfway between two trees in the plantation row) were randomly collected in each plot. The five single samples were homogenized with the utilization of a sterile bottle of glass to make one composite sample.

Subsamples from each composite sample were immediately placed in centrifuge tubes in the field and were stored in liquid nitrogen for the molecular analyses. Another subsample, destined for mineral nitrogen analysis, was held in a glass bottle and stored at 4°C until analysis. The remaining soil was air dried, stored in plastic bags, and used for physical and chemical analyses.

This study used the same soil samples screened for physico-chemical and biological properties described in a published work [25]. Thus, the information about the physical and chemical characteristics of the sites has been previously described and discussed, and the general soil characterization can be found in S1 Table.

### Fungal community structure analysis

The soil DNA was extracted using a PowerSoil DNA Isolation Kit (Mobio, CA, USA), according to the manufacturer’s instructions (except for the lysis step, which was modified to use FastPrep equipment (Bio 101, CA, USA) at a frequency of 5.5 for 40 seconds).

The fungal communities were studied using pyrosequencing [26]. The extracted DNA was subjected to PCR amplification targeting a fragment of the large sub-unit of the rRNA gene (28S rRNA) using the MID adapted primers (barcodes with 10 nucleotides added to each primer) LR0R (5′-ACCCGCTGAACTTAAGC-3′) [27] and LR3 (5′-CCGTGTTTCAAGACGGG-3′) [28]. The primers amplify the region 26 to 651 of the 28S rRNA (in relation to *Saccharomyces cerevisiae*). PCR reactions of 20 μl containing Buffer 1X, MgCl_2_ 3.37 mM, dNTP 5 nM each, BSA 0.1 mg ml^-1^ and GoTaq 2.5 μ (Promega) were performed in triplicate per sample, with the following program: 94°C for 3 min; followed by 30 cycles of 94°C for 1 min, 51°C for 40 s and 72°C for 1 min; and a final extension step of 72°C for 10 min. After amplification, the triplicate PCR reactions of each sample were combined, analyzed and extracted in 1.6% agarose gels, and subsequently purified in two steps using the QIAquick Gel Extraction and QIAquick PCR product purification kits (Qiagen). Equimolar amounts of the PCR products from the different samples were combined and submitted to pyrosequencing on a Genome Sequencer Titanium system (454 Life Sciences, USA) at the Utah State University facility and were sequenced after a ligation step to insert adaptors into the amplicons. The sequences from the 24 samples are available at the NCBI Sequence Read Archive under the following accession numbers: SRS503403, SRS503405, SRS503406 and from SRS503410 to SRS503430.

Raw sequences were processed through the Ribosomal Database Project (RDP) pyrosequencing pipeline (http://pyro.cme.msu.edu). Sequences were excluded from the analysis if one or more of the following conditions was not fulfilled: high quality (Q>20), read length higher than 200 nucleotides, absence of ambiguous bases, and presence of primer and barcode sequence.

The high quality sequences were randomly normalized to the same number of sequences using Mothur v1.26 [29] and submitted to the RDP-II classifier to obtain the taxonomic assignment and the relative abundance of the different fungal groups [30,31]. The classifier was run at a 50% confidence threshold to determine the identification of the fungal community and at 0% to generate the matrix for statistical analysis. All sequences that did not belong to kingdom Fungi were discarded. To increase the reliability of the results, we considered at any taxonomic level only the taxa represented by more than 10 sequences (minimum frequency of 0.02%).

From 24 samples, 22 could be normalized to 2.3 K sequences per sample. Two other samples had a lower number of sequences (0.6 K and 1 K). They were retained in some analyses (determination of the relative abundance and multidimensional ordinations) that are not biased by the number of sequences and were excluded from others (diversity estimators) that are strongly affected by the number of sequences. In this case, the other treatments also had two samples excluded to keep the same number of replicates per treatment.

### Data analysis

The taxonomic assignment was used to construct a matrix for each taxonomic level (phyla and genera). Each matrix was ordinated using NMS [32,33] with the Sørensen distance [34] and a random initial configuration. The significance of the matrix data structure was assessed using a Monte Carlo test. For ordinations, a secondary matrix was used to overlay the major gradients of soil chemical properties (Pearson’s correlation coefficient with p<0.05), allowing the direct assessment of the relationship between soil variables and fungal community structure. Although all variables in S1 Table were present in the ordination analysis, only those that significantly correlated with the microbial ordination are presented.

To confirm the existence of the groupings generated using NMS analysis, we performed a blocked Multi-Response Permutation Procedure **(**MRPP), which tests the hypothesis that no difference exists between two or more groups of entities [35]. To determine if the treatments had a significant effect on the specific groups of fungi, we used the blocked Indicator Species Analysis (ISA) [36]. The NMS ordination, MRPP and the ISA analyses were performed using the PC-ORD statistical package, V6.04.

The differences in the relative frequencies of phyla among treatments were tested using a blocked analysis of variance (ANOVA) followed by Tukey’s test.

To calculate the diversity indices, a matrix with distribution of the genera was processed using the statistical program PAST V2.17b [37], and the results were analyzed using Statistica V10 (StatSoft).

## Results and Discussion

From the three treatments, more than 50,000 high quality sequences were obtained, analyzed, and classified into more than 260 different genera. The rarefaction curve (S1 Fig.) showed that the sequencing depth covered most of the community. The soil fungal community did not vary significantly from the second to the third year after establishment in any of the treatments, which means that in the second year of the stands, the effects of the plants on the fungal community were already well established in a stable structure. Because there was no time effect, the data from the second and third year of cultivation are presented combined.

**Fig 1:**
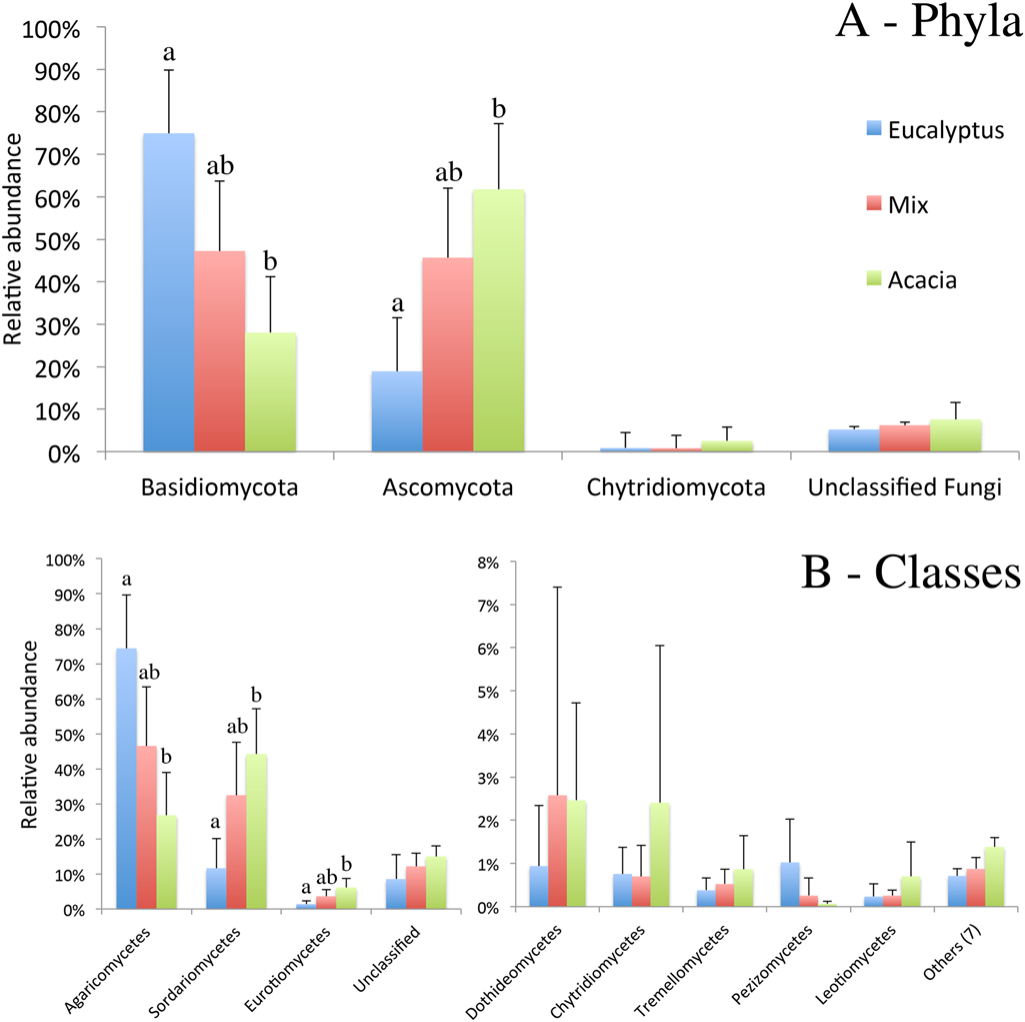
The relative frequencies of the different taxa found in the treatments Eucalyptus (monospecific stands of *Eucalyptus urograndis*), Acacia (monospecific stands of *Acacia mangium*) and Mix (intercropped plantation of these two species). The bars represent the average frequency (n = 8) and the error bars are the standard deviation. A. Phylum level; B. Class level. Different letters indicate significant differences among treatments in each taxon according to Tukey’s test (p<0.05).

The community composition at the phylum level was very distinct between the Eucalyptus and Acacia treatment, with a strong and significant effect of the plant species. However, the composition of the Mix treatment did not vary significantly from the monocultures, presenting an intermediate composition (Fig. 1A). A clear alternation in the relative abundances of the phyla Ascomycota and Basidiomycota was observed in soils under the influence of Eucalyptus or Acacia. The phylum Basidiomycota was the most abundant in Eucalyptus, corresponding on average to 75% of the community, whereas the phylum Ascomycota was the most frequent in Acacia, corresponding on average to 62% of the community. The Mix treatment had, respectively, 46% and 47% of these two phyla, showing equilibrium between the dominant phyla.

The phylum Chytridiomycota was also detected in all treatments, but at a lower frequency (average of 2.5% in Acacia and 0.8% in Eucalyptus and Mix). In addition, some sequences belonging to the phyla Glomeromycota and Blastocladiomycota were found at frequencies lower than 0.1%.

Another study that used the same 454 pyrosequencing system screened the ITS region to study the soil fungal community in soils with four genera from the family Pinaceae and two from the family Fagaceae determined that the phylum Basidiomycota was the most abundant in soils from all tree species studied [38]. However, the level of dominance varied according to the tree species at the sites, ranging from 65% (pine) to 28% (spruce), with unclassified Dikarya or Ascomycota as the second most abundant phyla. In contrast with our study, no sequences belonging to Chytridiomycota were described, and the Ascomycota phylum was not found in high abundance compared with the other phyla.

At the class level, the three most abundant classes were Agaricomycetes, Sordariomycetes and Eurotiomycetes. The first one was more abundant in Eucalyptus than in Acacia, whereas the other two were more frequent in Acacia than in Eucalyptus (Fig. 1B). The Mix treatment presented a relative abundance of these classes that was not significantly different from the monoculture treatments. Several other classes were also detected, but in lower frequencies and higher variability among replicates, with no significant differences among the treatments (Fig. 1B).

**Fig 2:**
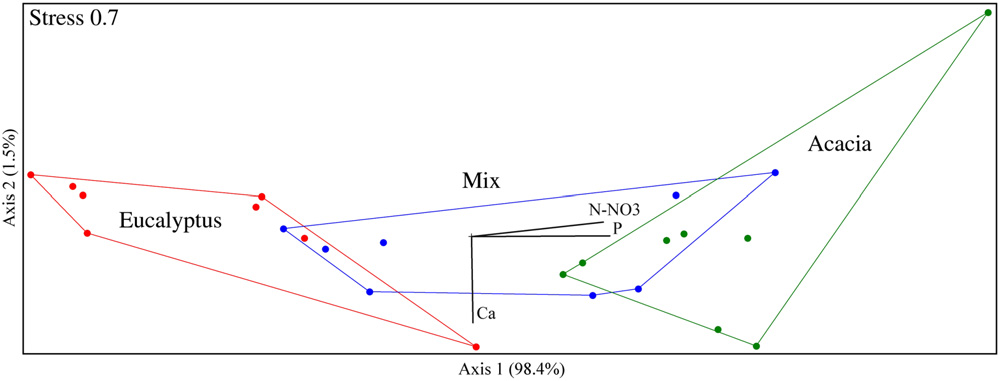
NMS ordination of the pyrosequencing data at the phylum level. The stress is in the scale of 0 to 100.

The ordination of the data based on the relative abundance of the groups in each treatment showed a strong effect of the treatments on the fungal community structure at the phylum level (Fig. 2), with significant differentiation among all treatments (MRPP<0.05). The same structure and the significant differentiation were present at the genera level, however, with higher variability within each treatment (data not shown).

Rachid et al. [25] evaluated the bacterial community structure from DGGE profiles in samples from the same stands and observed a similar result, with a distinct bacterial community structure in the monocultures and an intermediate structure in the mixed plantation.

There was a significant correlation of the available phosphorus and soil nitrate content with Axis 1 of the ordination, which separated the fungal community structure in treatments. Calcium correlated with Axis 2 and seemed to be associated with variations within replicates of each treatment. Fungi are of great importance for phosphorus solubilization in soils, as well as for N cycling. Recently, some studies have demonstrated that, under intercropping systems, some mycorrhizal fungi could improve nodulation, biological nitrogen fixation, P uptake and nitrogen transfer between plants [39–42]. Therefore, changes in the structure of the fungal community could have impacted the availability of soil phosphorus and nitrate contents, resulting in higher amounts of available P and nitrate in the Acacia and Mix treatments. It is important to note that the higher levels of N were found in the treatments without N fertilization. Additionally, the differences in these nutrient levels could impact the fungal community. The same samples had different numbers of gene copies related with nitrification (*amoA*) and denitrification (*nirK*), as seen in a previous study [25], which could also help to explain the differences in the nitrate content.

**Fig 3:**
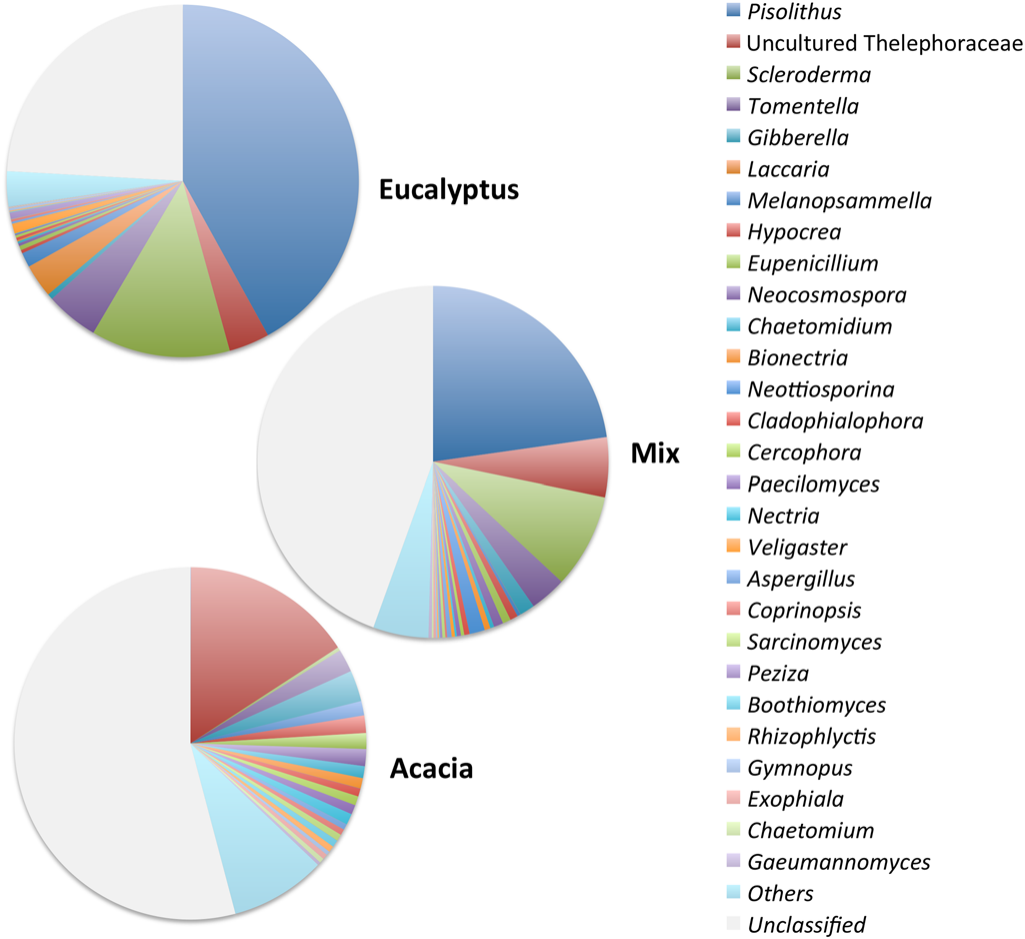
The relative frequencies (average; n = 8) of the 28 most frequent genera found in the treatments Eucalyptus (monospecific stands of *Eucalyptus urograndis*), Acacia (monospecific stands of *Acacia mangium*) and Mix (intercropped plantation of these two species). The blank part of each pie chart represents the unclassified sequences.

The influence of the plant species is even more evident when the fungal community is analyzed at the genus level (Fig. 3). For example, in the Eucalyptus treatment, an average of 42% of the sequences belonged to the genus *Pisolithus*, and approximately 13% belonged to the genus *Scleroderma*. *Pisolithus* is commonly associated with *Eucalyptus* and can be used as an inoculant to improve *Eucalyptus* development [22]. Despite their previously described occurrence with *Acacia mangium* [43], *Pisolithus* and *Scleroderma* did not occur at a significant frequency in the Acacia treatment. In contrast, one genus of an uncultured Thelephoraceae accounted for 16% of the sequences in Acacia samples but accounted for only 4% in Eucalyptus. Very few fungal genera showed a cosmopolitan distribution. One exception was the genus *Tomentella*, which was present in all samples in a high and constant abundance.

The species indicator analysis showed that 17 of the 25 genera were significantly related with one specific treatment, with four of them related with Eucalyptus (*Pisolithus*, *Scleroderma*, *Laccaria* and *Veligaster*) and 13 related with Acacia (uncultured Thelephoraceae, *Gibberella*, *Melanopsammella*, *Eupenicillium*, *Neocosmospora*, *Hypocrea*, *Nectria*, *Cercophora*, *Karlingi*, *Chaetomidium*, *Cladophialophora*, *Bionectria* and *Paecilomyces*). There were no genera related specifically with the Mix treatment, which means that the all genera present in high abundance in the Mix treatment had also a high abundancy in Eucalyptus or in Acacia. A list of the top 150 genera found in the three treatments can be found in S2 Table.

The plant species also affected significantly the fungal diversity indices of the systems (Table 1). The number of genera and the Shannon index were higher in the Acacia and Mix treatments in comparison with Eucalyptus; the Evenness index was also higher in Acacia than Eucalyptus, but the Mix treatment did not differ from both monocultures. Conversely, the dominance was significantly higher in Eucalyptus than in Acacia. The first four more frequent genera accounted on average for 84% of all classified sequences in Eucalyptus, 72% in the Mix and 46% in Acacia. The less frequent genera (those excluding the top 28) accounted for just 5% of the classified sequences in Eucalyptus, 10% in the Mix and 19% in Acacia. As reviewed by Chaparro and colleagues [44], the evenness has been identified as an important factor in community functioning, soil health, and plant productivity. According to their assessment, reduced dominance implies increased soil function and stability, which in turn could influence nutrient cycling and productivity.

**Table 1:** Diversity indexes of the treatments.

	Eucalyptus	Mix	Acacia
Number of genera	116 (19) b	145 (24) a	161 (8) a
Shannon H	2.37 (0.54) b	3.12 (0.54) a	3.68 (0.36) a
Dominance D	0.26 (0.16) a	0.15 (0.07) ab	0.08 (0.05) b
Evenness	0.10 (0.04) b	0.17 (0.07) ab	0.26 (0.08) a

The numbers are averages of 6 replicates per treatment, with the standard deviations inside parenthesis. Numbers followed by different letters in the row are significantly different (Tukey’s, p<5%).

The results clearly demonstrate the effect of plants on the soil fungal community. Despite the homogeneity of soil properties and the proximity of the plots, the two treatments with monoculture (Acacia and Eucalyptus) showed very distinct community structures, compositions and diversities. When the two plant species were intercropped, the characteristics of the fungal community from each monoculture were integrated, forming an intermediate state.

The pyrosequencing approach allowed the identification of the major groups affected by each plant species, allowing the inference of the mechanism by which the community was affected. Plants can influence microorganisms through root exudates, rhizodeposits and litter fall, modulating the microbial community, potentially attracting microorganisms able to supply nutritional deficits (*i*.*e*., N, P) and/or help in the defense against pathogens and invader plants and insects [42,44–46]. The most abundant genera found in this work can establish ectomycorrhizal associations, with hosts such as *Eucalyptus* and *Acacia* [42,43,45–47]. It is known that many ectomycorrhizal associations are species-specific; that is, which plants can attract a given fungal group through chemical signaling mechanisms and physiological compatibility [48]. Therefore, most likely due to these associations, each tree species has selected for some particular groups of fungi.

According to Terdesoo et al. [49] is not clear whether or not richness of host plants affects local ectomycorrhyzal fungi species richness in natural environments. It probably depends on a number of different factors, which include the size and dispersion capacity of the fungal species pool present in the environment, as well as soil properties and plant distribution. Plant richness *per se* does not explain the variation of the richness of ectomycorrhyzal fungi species in complex enviroment. However,Terdesoo and coleagues highlight that there is a strong effect of particular plant species in shaping the community of ectomycorrhyzal fungi, what is called ‘host effect’. This effect would be more intense in sites with low plant diversity, such as the present study (monospecific stands or two species stands). This was also observed in another study with members of the plant family Salicaceae, which showed that phylogeny of the plant host could explain 75% of the variation in species richness and 20% of the variation in community composition of ectomycorrhyzal fungi [50].

Since the soil had not been sampled before the treatment establishment, we could not determine whether the fungal genera were residents in the soil or if they were introduced with the seedlings. Most of the genera were found in all treatments, and probably were present in the soil before the experiment introduction. However, the genus *Pisolithus* was found just in half of the samples in the Acacia, and in very low abundances. In contrast, *Pisolithus* was very abundant in Eucalyptus and Mix treatments, indicating that it could have been introduced with the *Eucalyptus* seedlings. The genus *Laccaria*, on the other hand, probably was suppressed by Acacia, since it could only be found in one sample of the Mix treatment and could not be detected in the Acacia treatment.

The very low incidence of arbuscular mycorrhizae was unexpected as its occurrence was previously reported in association with both plant species, sometimes in higher frequency than ectomycorrizae [21,23, 24]. Is very unlikely that the absence of arbuscular mycorrhizal fungi has been caused by primer bias, since the primer was complementary to sequences from all genera belonging to Glomeromycota according to the RDP Probe Match tool. Possibly the successful colonization of roots by ectomycorrhizal fungi inhibited the development of arbuscular mycorrhizal fungi, due to competition.

The establishment of the fungal community most likely followed the root dynamics. Because above and below ground competition between trees is high before and during the closure of the canopy, a fast horizontal and vertical occupation of soil volume by roots happens in the early stages of the plant development [11,17,51]. Silva et al. [52] observed a fast fine root occupation and dynamics under pure and mixed plantations of *Eucalyptus grandis* and *Acacia mangium* up to 30 months after planting, which supports the early establishment of the soil fungal community.

Our data suggest that management of the soil fungal community is possible. Through the introduction of the tree species *Acacia mangium* into a *Eucalyptus* plantation, we significantly increased the number of genera of fungi, the diversity index and the frequency of several genera that were not found in the monoculture treatments. The intercropped systems could be a key to achieving soil microbial management, as a powerful biotechnological tool. Some microbial groups of great interest could be introduced into the soil community by the association of two plant species, one of economic importance and another of ecological importance, that select or harbor the desired microbial group(s). More research with different plant groups under different ecological conditions is needed to assess the broader value of this approach.

## Supporting Information

S1 Fig Rarefaction curve of the samples from the three treatments. (PDF)

S1 Table Chemical characterization of the bulk soil samples (0–10cm) from the three treatments at the second and third year of the plantation. (PDF)

S2 Table Relative frequency of the top 150 genera in the three treatments. The percentages are the averages (n = 8) of the classified sequences belonging to each genus and the standard deviations.
(PDF)
