# Supplementary figures and images for: Intercropped Silviculture Systems, a Key to Achieving Soil Fungal Community Management in Eucalyptus Plantations
Source: PLoS One. 2015 Feb 23;10(2):e0118515. doi: 10.1371/journal.pone.0118515 (PMC4338270; doi:10.1371/journal.pone.0118515)

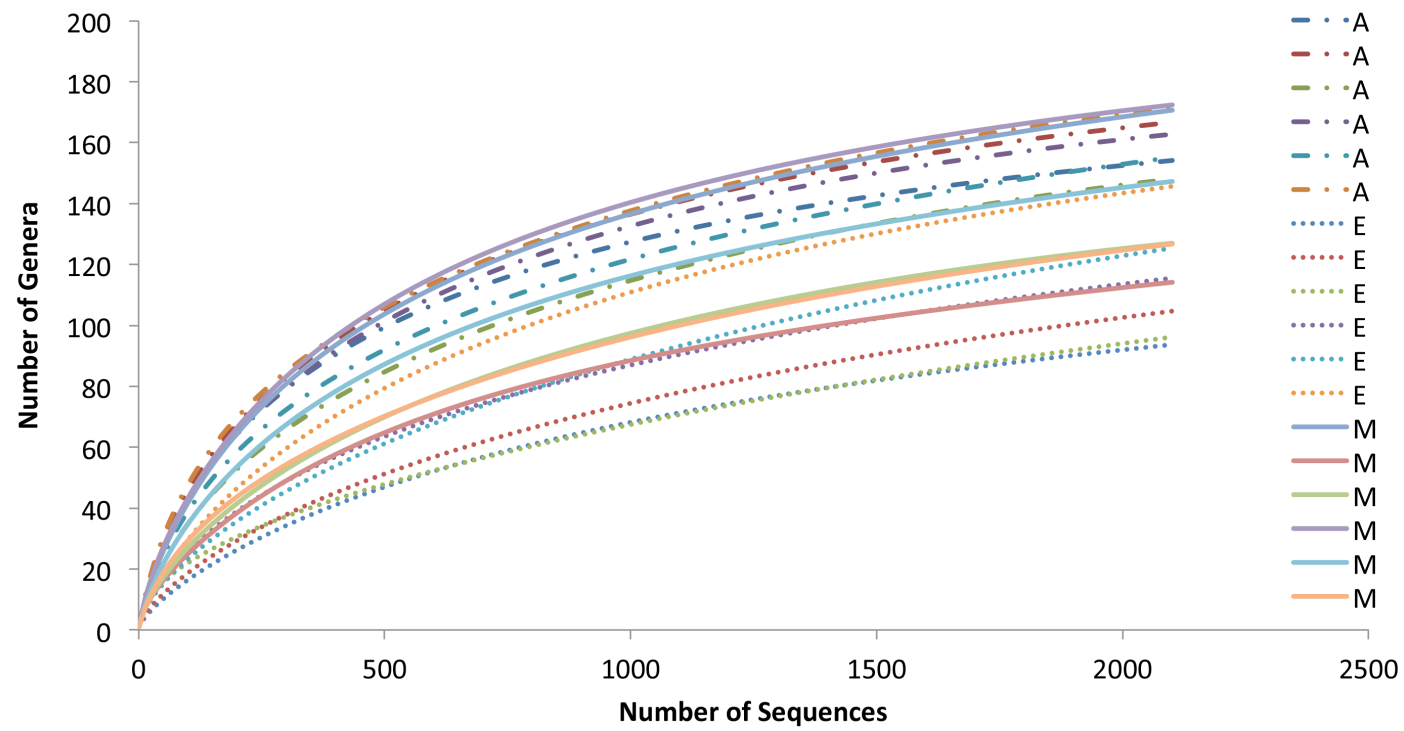

Figure S1 – Rarefaction curve of the samples from the three treatments.

Supplement: S1 Fig — (PDF) [file pone.0118515.s001.pdf]
