# Supplementary material for: Intercropped Silviculture Systems, a Key to Achieving Soil Fungal Community Management in Eucalyptus Plantations
Source: PLoS One. 2015 Feb 23;10(2):e0118515. doi: 10.1371/journal.pone.0118515 (PMC4338270; doi:10.1371/journal.pone.0118515)
Supplement: S1 Table — (PDF) [file pone.0118515.s002.pdf]

Table S1 – Chemical characterization of the bulk soil samples (0-10cm) from the three treatments at the second and third year of the plantation.

	Eucalyptus		Mix		*Acacia*	
	2nd year	3rd year	2nd year	3rd year	2nd year	3rd year
pH	5.45(0.13)	5.73(0.25)	5.40(0.20)	5.48(0.13)	5.38(0.13)	5.88(0.14)
Total C	4.49(0.42)	6.43(0.65)*	4.43(0.32)	5.38(0.62)*	4.15 (0.34)	7.38(0.83)*
Total N	0.35(0.04)	0.51(0.05)*	0.34(0.04)	0.44(0.05)*	0.32(0.03)	0.58(0.06)*
C:N	12.85(0.16)	12.72(0.52)	13.09(0.22)	12.06(0.18)	12.92(0.11)	12.76(0.27)
$N\text{-}NH_4$	1.50(0.79)	1.68(0.35)	1.62(0.53)	1.21(0.08)	2.07(0.58)	1.29(0.29)
	a		b		b	
$N\text{-}NO_3$	0.06(0.06)	0.35(0.35)	0.06(0.04)	1.65(0.71)*	0.48(0.18)	2.75(0.97)*
Exchangeable Al	0.08(0.03)	0.08(0.03)	0.08(0.03)	0.08(0.05)	0.13(0.03)	0.03(0.03)
Exchangeable Ca	0.93(0.14)	1.08(0.19)	0.90(0.23)	1.20(0.27)	0.80(0.17)	1.05(0.09)
Exchangeable Mg	1.20(0.09)	0.78(0.08)	1.00(0.14)	1.00(0.11)	1.03(0.06)	0.93(0.20)
Exchangeable Na	1.25(1.25)a	14.00(0.82)*	1.25(1.25)	13.50(0.50)*	0.50(0.50)	11.00(1.22)*
Exchangeable K	22.50(2.72)	24.50(2.72)	28.50(9.21)	38.00(10.12)	24.25(4.78)	30.25(5.09)
Exchangeable H+Al	2.38(0.43)	2.08(0.23)	2.53(0.22)	2.23(0.11)	2.18(0.22)	1.85(0.13)
	a		b		b	
Available P	9.30(2.20)	8.95(1.97)	14.58(3.11)	13.05(3.12)	13.28(2.17)	14.55(2.28)
Sums of Bases	2.18(0.18)	1.95(0.27)	1.98(0.41)	2.35(0.39)	1.90(0.25)	2.10(0.31)
CEC	4.58(0.46)	4.08(0.08)	4.55(0.38)	4.58(0.38)	4.10(0.44)	3.95(0.38)
V	48.75(4.55)	48.00(5.96)	42.75(5.44)	37.65(11.44)	46.00(2.04)	52.75(2.95)

*Significant difference between second and third year within treatment. Letters over Total P values represent the differentiation among treatments according to Tukey test (5%). The numbers represent average values (n=4) with each standard error inside brackets. Parameters units: Al, Ca, Mg, H+Al, P, SB, CEC ($cmol_c\ dm^{-3}$), Na, K ($mg\ dm^{-3}$), V (%), C, N ($g\ Kg^{-1}$).
