# Supplementary material for: Intercropped Silviculture Systems, a Key to Achieving Soil Fungal Community Management in Eucalyptus Plantations
Source: PLoS One. 2015 Feb 23;10(2):e0118515. doi: 10.1371/journal.pone.0118515 (PMC4338270; doi:10.1371/journal.pone.0118515)
Supplement: S2 Table — The percentages are the averages (n = 8) of the classified sequences belonging to each genus and the standard deviations. (PDF) [file pone.0118515.s003.pdf]

Table S2 – Relative frequency of the top 150 genera in the three treatments. The percentages are the averages (n=8) of the classified sequences belonging to each genus and the standard deviations

| Genus | Eucalyptus | | Mix | | Acacia | |
|---|---|---|---|---|---|---|
| | **Average** | Std Deviation | **Average** | Std Deviation | **Average** | Std Deviation |
| *Pisolithus* | **42.0%** | 15.6% | **22.8%** | 19.1% | **0.0%** | 0.1% |
| uncultured Thelephoraceae | **3.7%** | 2.6% | **5.5%** | 5.6% | **15.8%** | 11.1% |
| *Scleroderma* | **12.8%** | 7.4% | **8.7%** | 5.4% | **0.3%** | 0.8% |
| *Tomentella* | **4.9%** | 4.0% | **3.4%** | 2.9% | **2.1%** | 2.2% |
| *Gibberella* | **0.5%** | 0.6% | **1.4%** | 1.0% | **2.9%** | 1.4% |
| *Laccaria* | **3.1%** | 5.1% | **0.0%** | 0.0% | **0.0%** | 0.0% |
| *Melanopsammella* | **1.3%** | 3.4% | **0.3%** | 0.4% | **1.3%** | 1.5% |
| *Hypocrea* | **0.3%** | 0.3% | **0.7%** | 0.3% | **1.6%** | 1.0% |
| *Eupenicillium* | **0.4%** | 0.3% | **0.7%** | 0.6% | **1.5%** | 0.8% |
| *Neocosmospora* | **0.3%** | 0.3% | **0.9%** | 0.7% | **1.6%** | 1.3% |
| *Chaetomidium* | **0.2%** | 0.2% | **0.4%** | 0.2% | **1.1%** | 0.7% |
| *Bionectria* | **0.1%** | 0.1% | **0.5%** | 0.5% | **1.0%** | 0.6% |
| *Neottiosporina* | **0.0%** | 0.0% | **1.4%** | 4.0% | **0.0%** | 0.0% |
| *Cladophialophora* | **0.2%** | 0.3% | **0.5%** | 0.4% | **0.7%** | 0.5% |
| *Cercophora* | **0.2%** | 0.1% | **0.3%** | 0.2% | **0.8%** | 0.6% |
| *Paecilomyces* | **0.1%** | 0.1% | **0.4%** | 0.5% | **0.9%** | 1.2% |
| *Nectria* | **0.1%** | 0.1% | **0.2%** | 0.2% | **1.0%** | 0.6% |
| *Veligaster* | **0.9%** | 1.3% | **0.3%** | 0.2% | **0.0%** | 0.0% |
| *Aspergillus* | **0.2%** | 0.2% | **0.4%** | 0.3% | **0.5%** | 0.4% |
| *Coprinopsis* | **0.2%** | 0.3% | **0.2%** | 0.2% | **0.5%** | 0.5% |
| *Sarcinomyces* | **0.0%** | 0.0% | **0.3%** | 0.3% | **0.6%** | 0.5% |
| *Peziza* | **0.6%** | 0.7% | **0.2%** | 0.4% | **0.0%** | 0.0% |
| *Boothiomyces* | **0.1%** | 0.1% | **0.1%** | 0.1% | **0.6%** | 1.1% |
| *Rhizophlyctis* | **0.1%** | 0.1% | **0.1%** | 0.2% | **0.6%** | 1.4% |
| *Gymnopus* | **0.2%** | 0.4% | **0.1%** | 0.2% | **0.4%** | 0.9% |
| *Exophiala* | **0.0%** | 0.1% | **0.2%** | 0.2% | **0.5%** | 0.4% |
| *Chaetomium* | **0.0%** | 0.0% | **0.2%** | 0.1% | **0.4%** | 0.4% |
| *Gaeumannomyces* | **0.1%** | 0.1% | **0.3%** | 0.3% | **0.3%** | 0.3% |
| *Cyathus* | **0.5%** | 1.5% | **0.0%** | 0.0% | **0.0%** | 0.0% |
| *Westerdykella* | **0.1%** | 0.0% | **0.2%** | 0.2% | **0.3%** | 0.2% |
| *Dipodascopsis* | **0.1%** | 0.2% | **0.1%** | 0.1% | **0.3%** | 0.2% |
| *Cryptadelphia* | **0.1%** | 0.2% | **0.2%** | 0.1% | **0.2%** | 0.2% |
| *Bourdotia* | **0.1%** | 0.1% | **0.2%** | 0.4% | **0.2%** | 0.2% |
| *Mycena* | **0.1%** | 0.1% | **0.1%** | 0.1% | **0.2%** | 0.2% |
| *Lanspora* | **0.0%** | 0.1% | **0.1%** | 0.1% | **0.3%** | 0.2% |
| *Ophiostoma* | **0.0%** | 0.0% | **0.1%** | 0.1% | **0.3%** | 0.2% |
| *Xylaria* | **0.0%** | 0.0% | **0.1%** | 0.2% | **0.3%** | 0.8% |
| *Cryptococcus* | **0.1%** | 0.1% | **0.1%** | 0.1% | **0.2%** | 0.3% |
| *Immersiella* | **0.0%** | 0.0% | **0.2%** | 0.5% | **0.2%** | 0.5% |
| *Papulosa* | **0.1%** | 0.1% | **0.1%** | 0.1% | **0.2%** | 0.1% |
| *Pochonia* | **0.0%** | 0.0% | **0.1%** | 0.1% | **0.3%** | 0.3% |

| | | | | | | |
|---|---|---|---|---|---|---|
| *Chromocleista* | **0.0%** | 0.1% | **0.1%** | 0.1% | **0.3%** | 0.1% |
| *Pyrenochaeta* | **0.1%** | 0.1% | **0.1%** | 0.1% | **0.2%** | 0.3% |
| *Coniochaeta* | **0.0%** | 0.1% | **0.1%** | 0.1% | **0.2%** | 0.1% |
| *Stachybotrys* | **0.1%** | 0.2% | **0.3%** | 0.5% | **0.0%** | 0.0% |
| *Entorrhiza* | **0.1%** | 0.1% | **0.1%** | 0.1% | **0.2%** | 0.2% |
| *Thelephora* | **0.0%** | 0.0% | **0.1%** | 0.1% | **0.3%** | 0.2% |
| *Tremella* | **0.0%** | 0.0% | **0.2%** | 0.1% | **0.1%** | 0.1% |
| *Lycoperdon* | **0.0%** | 0.1% | **0.1%** | 0.1% | **0.2%** | 0.4% |
| *Penicillium* | **0.0%** | 0.0% | **0.1%** | 0.2% | **0.2%** | 0.1% |
| *Phialophora* | **0.1%** | 0.1% | **0.2%** | 0.3% | **0.2%** | 0.2% |
| *Neurospora* | **0.0%** | 0.0% | **0.1%** | 0.1% | **0.2%** | 0.4% |
| *Melanospora* | **0.0%** | 0.0% | **0.1%** | 0.1% | **0.2%** | 0.2% |
| *Hydropus* | **0.0%** | 0.0% | **0.2%** | 0.4% | **0.1%** | 0.3% |
| *Chaetosphaeria* | **0.1%** | 0.2% | **0.0%** | 0.1% | **0.1%** | 0.4% |
| *Gelasinospora* | **0.0%** | 0.0% | **0.1%** | 0.1% | **0.2%** | 0.3% |
| *Mycetophylax* | **0.1%** | 0.1% | **0.0%** | 0.1% | **0.1%** | 0.1% |
| *Pestalotiopsis* | **0.0%** | 0.0% | **0.0%** | 0.0% | **0.2%** | 0.1% |
| *Lasiodiplodia* | **0.0%** | 0.0% | **0.1%** | 0.1% | **0.2%** | 0.2% |
| *Nigrospora* | **0.0%** | 0.0% | **0.1%** | 0.1% | **0.1%** | 0.2% |
| *Rhizophydium* | **0.1%** | 0.2% | **0.0%** | 0.1% | **0.1%** | 0.1% |
| *Panaeolus* | **0.2%** | 0.4% | **0.0%** | 0.0% | **0.0%** | 0.0% |
| *Sorocybe* | **0.0%** | 0.0% | **0.0%** | 0.0% | **0.1%** | 0.1% |
| *Terfezia* | **0.1%** | 0.2% | **0.0%** | 0.0% | **0.0%** | 0.0% |
| *Lophiostoma* | **0.1%** | 0.4% | **0.0%** | 0.0% | **0.0%** | 0.0% |
| *Zygopleurage* | **0.0%** | 0.0% | **0.0%** | 0.0% | **0.1%** | 0.3% |
| *Atractiella* | **0.0%** | 0.0% | **0.0%** | 0.0% | **0.2%** | 0.4% |
| *Chlorophyllum* | **0.0%** | 0.0% | **0.1%** | 0.1% | **0.1%** | 0.1% |
| *Heterochaete* | **0.0%** | 0.0% | **0.0%** | 0.0% | **0.2%** | 0.4% |
| *Cordyceps* | **0.0%** | 0.0% | **0.1%** | 0.1% | **0.1%** | 0.1% |
| *Monascus* | **0.0%** | 0.0% | **0.2%** | 0.2% | **0.0%** | 0.0% |
| *Scolecobasidiella* | **0.0%** | 0.0% | **0.0%** | 0.0% | **0.1%** | 0.1% |
| *Fibulobasidium* | **0.1%** | 0.1% | **0.0%** | 0.0% | **0.1%** | 0.1% |
| *Lentomitella* | **0.0%** | 0.0% | **0.0%** | 0.0% | **0.1%** | 0.3% |
| *Arachnomyces* | **0.0%** | 0.0% | **0.0%** | 0.1% | **0.1%** | 0.2% |
| *Plectosphaerella* | **0.0%** | 0.0% | **0.0%** | 0.0% | **0.1%** | 0.1% |
| *Eurotium* | **0.0%** | 0.0% | **0.0%** | 0.1% | **0.1%** | 0.1% |
| *Vararia* | **0.0%** | 0.0% | **0.0%** | 0.0% | **0.1%** | 0.3% |
| *Aniptodera* | **0.0%** | 0.0% | **0.0%** | 0.1% | **0.0%** | 0.1% |
| *Porosphaerellopsis* | **0.0%** | 0.1% | **0.0%** | 0.1% | **0.0%** | 0.1% |
| *Corynascus* | **0.0%** | 0.0% | **0.1%** | 0.1% | **0.1%** | 0.0% |
| *Cochliobolus* | **0.0%** | 0.0% | **0.0%** | 0.0% | **0.1%** | 0.1% |
| *Asterostroma* | **0.0%** | 0.0% | **0.0%** | 0.1% | **0.0%** | 0.1% |
| *Chytridium* | **0.1%** | 0.1% | **0.0%** | 0.0% | **0.0%** | 0.0% |
| *Ileodictyon* | **0.0%** | 0.0% | **0.1%** | 0.2% | **0.0%** | 0.0% |
| *Batistia* | **0.0%** | 0.0% | **0.1%** | 0.1% | **0.1%** | 0.1% |
| *Podospora* | **0.0%** | 0.0% | **0.0%** | 0.0% | **0.1%** | 0.1% |

| | | | | | | |
|---|---|---|---|---|---|---|
| *Microdiplodia* | **0.0%** | 0.0% | **0.0%** | 0.1% | **0.0%** | 0.1% |
| *Poromycena* | **0.0%** | 0.1% | **0.0%** | 0.0% | **0.0%** | 0.1% |
| *Moniliophthora* | **0.0%** | 0.0% | **0.1%** | 0.2% | **0.0%** | 0.0% |
| *Catenomyces* | **0.0%** | 0.0% | **0.0%** | 0.0% | **0.1%** | 0.1% |
| *Sarcostroma* | **0.0%** | 0.0% | **0.1%** | 0.3% | **0.0%** | 0.0% |
| *Troposporella* | **0.1%** | 0.1% | **0.0%** | 0.1% | **0.0%** | 0.0% |
| *Corynespora* | **0.0%** | 0.0% | **0.0%** | 0.1% | **0.0%** | 0.0% |
| *Gymnopilus* | **0.0%** | 0.0% | **0.0%** | 0.1% | **0.1%** | 0.1% |
| *Apiosordaria* | **0.0%** | 0.0% | **0.0%** | 0.0% | **0.0%** | 0.0% |
| *Anthostomella* | **0.0%** | 0.0% | **0.0%** | 0.0% | **0.0%** | 0.1% |
| *Spizellomyces* | **0.0%** | 0.0% | **0.0%** | 0.0% | **0.0%** | 0.1% |
| *Kappamyces* | **0.0%** | 0.0% | **0.0%** | 0.1% | **0.0%** | 0.0% |
| *Basidiodendron* | **0.0%** | 0.0% | **0.0%** | 0.0% | **0.1%** | 0.1% |
| *Omphalina* | **0.0%** | 0.0% | **0.0%** | 0.0% | **0.0%** | 0.1% |
| *Marasmiellus* | **0.0%** | 0.0% | **0.0%** | 0.0% | **0.0%** | 0.1% |
| *Macrophomina* | **0.0%** | 0.0% | **0.0%** | 0.1% | **0.0%** | 0.0% |
| *Stagonospora* | **0.0%** | 0.0% | **0.0%** | 0.1% | **0.0%** | 0.0% |
| *Chaetocalathus* | **0.0%** | 0.0% | **0.1%** | 0.2% | **0.0%** | 0.0% |
| *Lecanicillium* | **0.0%** | 0.0% | **0.0%** | 0.0% | **0.0%** | 0.0% |
| *Phlyctochytrium* | **0.0%** | 0.0% | **0.0%** | 0.0% | **0.0%** | 0.0% |
| *Inocephalus* | **0.0%** | 0.0% | **0.0%** | 0.1% | **0.0%** | 0.1% |
| *Platygloea* | **0.0%** | 0.0% | **0.1%** | 0.1% | **0.0%** | 0.0% |
| *Triangularia* | **0.0%** | 0.0% | **0.0%** | 0.0% | **0.0%** | 0.0% |
| *Ophiocordyceps* | **0.0%** | 0.1% | **0.0%** | 0.0% | **0.0%** | 0.0% |
| *Thanatephorus* | **0.0%** | 0.0% | **0.0%** | 0.1% | **0.0%** | 0.0% |
| *Petriella* | **0.0%** | 0.0% | **0.0%** | 0.0% | **0.0%** | 0.1% |
| *Preussia* | **0.0%** | 0.0% | **0.0%** | 0.0% | **0.0%** | 0.0% |
| *Clavaria* | **0.0%** | 0.0% | **0.0%** | 0.0% | **0.0%** | 0.0% |
| *Piriformospora* | **0.0%** | 0.0% | **0.0%** | 0.0% | **0.0%** | 0.0% |
| *leaf* | **0.0%** | 0.0% | **0.0%** | 0.0% | **0.0%** | 0.0% |
| *Rhodoveronaea* | **0.0%** | 0.1% | **0.0%** | 0.0% | **0.0%** | 0.0% |
| *Metarhizium* | **0.0%** | 0.0% | **0.0%** | 0.0% | **0.0%** | 0.0% |
| *Metacordyceps* | **0.0%** | 0.0% | **0.0%** | 0.0% | **0.0%** | 0.0% |
| *Cyphellophora* | **0.0%** | 0.0% | **0.0%** | 0.0% | **0.0%** | 0.0% |
| *Didymella* | **0.0%** | 0.0% | **0.0%** | 0.0% | **0.0%** | 0.0% |
| *Psathyrella* | **0.0%** | 0.0% | **0.0%** | 0.0% | **0.0%** | 0.0% |
| *Gastrocybe* | **0.0%** | 0.0% | **0.0%** | 0.0% | **0.0%** | 0.1% |
| *Agaricus* | **0.0%** | 0.0% | **0.0%** | 0.1% | **0.0%** | 0.0% |
| *Blastocladiella* | **0.0%** | 0.0% | **0.0%** | 0.0% | **0.0%** | 0.0% |
| *Jugulospora* | **0.0%** | 0.0% | **0.0%** | 0.0% | **0.0%** | 0.0% |
| *Sordaria* | **0.0%** | 0.0% | **0.0%** | 0.0% | **0.0%** | 0.0% |
| *Bartalinia* | **0.0%** | 0.0% | **0.0%** | 0.0% | **0.0%** | 0.1% |
| *Ctenomyces* | **0.0%** | 0.0% | **0.0%** | 0.0% | **0.0%** | 0.0% |
| *Cladosporium* | **0.0%** | 0.0% | **0.0%** | 0.0% | **0.0%** | 0.1% |
| *Keissleriella* | **0.0%** | 0.0% | **0.0%** | 0.0% | **0.0%** | 0.0% |
| *Lophiotrema* | **0.0%** | 0.0% | **0.0%** | 0.0% | **0.0%** | 0.0% |

| | | | | | | |
|---|---|---|---|---|---|---|
| *Peyronellaea* | **0.0%** | 0.0% | **0.0%** | 0.0% | **0.0%** | 0.0% |
| *Quambalaria* | **0.0%** | 0.1% | **0.0%** | 0.0% | **0.0%** | 0.0% |
| *Cyrenella* | **0.0%** | 0.0% | **0.0%** | 0.0% | **0.0%** | 0.0% |
| *Asterotremella* | **0.0%** | 0.0% | **0.0%** | 0.0% | **0.0%** | 0.0% |
| *Agaricales* | **0.0%** | 0.0% | **0.0%** | 0.1% | **0.0%** | 0.0% |
| *Lepiota* | **0.0%** | 0.0% | **0.0%** | 0.0% | **0.0%** | 0.0% |
| *Clitopilus* | **0.0%** | 0.0% | **0.0%** | 0.0% | **0.0%** | 0.0% |
| *Tulasnella* | **0.0%** | 0.0% | **0.0%** | 0.0% | **0.0%** | 0.0% |
| *Pycnoporus* | **0.0%** | 0.0% | **0.0%** | 0.0% | **0.0%** | 0.1% |
| *Phaeomoniella* | **0.0%** | 0.0% | **0.0%** | 0.0% | **0.0%** | 0.0% |
| *Brachiosphaera* | **0.0%** | 0.0% | **0.0%** | 0.0% | **0.0%** | 0.0% |
| *Pichia* | **0.0%** | 0.0% | **0.0%** | 0.0% | **0.0%** | 0.0% |
| *Torulaspora* | **0.0%** | 0.0% | **0.0%** | 0.0% | **0.0%** | 0.1% |
| *Pulvinula* | **0.0%** | 0.0% | **0.0%** | 0.0% | **0.0%** | 0.0% |
| *Tainosphaeria* | **0.0%** | 0.0% | **0.0%** | 0.0% | **0.0%** | 0.0% |
| *Schizothecium* | **0.0%** | 0.0% | **0.0%** | 0.0% | **0.0%** | 0.0% |
| *Simplicillium* | **0.0%** | 0.0% | **0.0%** | 0.1% | **0.0%** | 0.0% |
